# Unusual Multiple Production of *N*-Acylhomoserine Lactones a by *Burkholderia* sp. Strain C10B Isolated from Dentine Caries

**DOI:** 10.3390/s140508940

**Published:** 2014-05-21

**Authors:** Share Yuan Goh, Wen-Si Tan, Saad Ahmed Khan, Hooi Pin Chew, Noor Hayaty Abu Kasim, Wai-Fong Yin, Kok-Gan Chan

**Affiliations:** 1 Division of Genetics and Molecular Biology, Institute of Biological Sciences, Faculty of Science, University of Malaya, 50603 Kuala Lumpur, Malaysia; E-Mails: shareyuan@um.edu.my (S.Y.G.); tmarilyn36@gmail.com (W.-S.T.); yinwaifong@yahoo.com (W.-F.Y.); 2 Department of Restorative Dentistry, Faculty of Dentistry, University of Malaya, 50603 Kuala Lumpur, Malaysia; E-Mails: saadkhan@um.edu.my (S.A.K.); chewhp@um.edu.my (H.P.C.); nhayaty@um.edu.my (N.H.A.K.)

**Keywords:** *Chromobacterium violaceum* CV026, *N*-hexanoyl-L-homoserine lactone (C6-HSL), *N*-octanoyl-L-homoserine lactone (C8-HSL), *N*-decanoyl-L-homoserine lactone (C10-HSL), *N*-dodecanoyl-L-homoserine lactone (C12-HSL), oral cavity, dentine caries, quorum sensing (QS)

## Abstract

Bacteria realize the ability to communicate by production of quorum sensing (QS) molecules called autoinducers, which regulate the physiological activities in their ecological niches. The oral cavity could be a potential area for the presence of QS bacteria. In this study, we report the isolation of a QS bacterial isolate C10B from dentine caries. Preliminary screening using *Chromobacterium violaceum* CV026 biosensor showed that isolate C10B was able to produce N-acylhomoserine lactones (AHLs). This bacterium was further identified as a member of *Burkholderia*, an opportunistic pathogen. The isolated *Burkholderia* sp. was confirmed to produce *N*-hexanoyl-L-homoserine lactone (C6-HSL), *N*-octanoyl-L-homoserine lactone (C8-HSL), *N*-decanoyl-L-homoserine lactone (C10-HSL) and *N*-dodecanoyl-L-homoserine lactone (C12-HSL).

## 1. Introduction

Recent advances in the study of bacterial gene expression have shed light on the topic of cell-to-cell communication and community behavior that are critical for successful interactions between higher organisms. Cell-to-cell communication, also known as quorum sensing (QS), acts as a tool that diversifies the physiological activities regulated by bacteria such as motility, biofilm formation and production of virulence factors [1]. The term QS, coined by Fuqua and colleagues, describes the cell-density dependent phenomenon whereby bacteria produce and respond to high intracellular concentrations of signal molecules inducing concerted physiological process in the bacterial community [2,3]. Once a critical threshold concentration population density has been achieved, the activation of a signal transduction cascade will drive the expression of target genes and allow a collective behavioral to occur [4,5]. QS molecules are also known as autoinducers and they vary in their structure [6].

QS mechanisms have evolved along three tracks, which include the *N*-acylhomoserine lactone (AHL)-based signaling system of Gram-negative bacteria, the oligopeptide-based system in Gram-positive bacteria and a shared furanone-based system that is common to both [7,8]. In most reports, AHL signals generally involve the LuxI family of AHL synthases and a LuxR family of transcriptional regulators [2]. The QS system in Gram-negative bacteria is typically dependent on a “LuxI” autoinducer synthase and a cognate “LuxR” transcriptional activator protein [9], whereby the binding of signal molecules perceived by a luxR protein will result in the activation of specific target genes under certain conditions, often at high cell population densities [10].

Most bacteria use QS to regulate numerous virulence determinants such as colonization and virulence factors indicating their pathogenicity that could cause a variety of human diseases [11] such as *Burkholderia cepacia*, an important pathogen in patients with cystic fibrosis often associated with bacteremia resulting in necrotizing pneumonia [3,12,13]. The presence of such pathogenic human bacteria has triggered interest in isolating and studying them. In view of this, we investigated the presence of *Burkholderia* sp. from dentine caries samples and report the isolation of these bacteria that display QS properties.

## 2. Experimental Section

### 2.1. Bacterial Strains and AHLs Preliminary Screening

*Chromobacterium violaceum* CV026 was used as short chain AHLs biosensor in this study. In the presence of short chain AHLs, *C. violaceum* CV026 responds by producing a purple violacein pigmentation [14,15]. In the screening, *Erwinia carotovora* GS101 and *E. carotovora* PNP22 were used as positive and negative controls, respectively [7,16,17]. All bacterial biosensor, positive and negative control strains were cultured routinely with Lysogeny broth (LB) medium (containing, in grams per 100 mL: tryptone, 1; yeast extract, 0.5; NaCl, 0.5; Bacto agar, 1.5) with 24 h incubation at 28 °C. Then we proceeded with preliminary screening of AHL production and routinely cultured bacteria on LB agar at 37 °C overnight (24 h). Medical ethics approval was obtained from the Dental Faculty University of Malaya Ethics and Research Committee (DFRD-1302/0033-L) prior to the start of the study. Informed consent was taken from a subject before tooth extraction at the Department of Oral and Maxillofacial Surgery, University of Malaya. Strain C10B was isolated from the dentine caries of the subject's extracted molar.

### 2.2. Caries Excavation and Microbiological Sampling

A freshly extracted molar with deep dentine caries that did not involve the pulp was used for this study. The patient was a healthy adult without any long-standing illness, hospitalization or communicable disease. The carious dentine was located within the coronal section of the tooth involving two surfaces. Unsupported enamel was removed using a sterile water-cooled diamond bur operated with an air-turbine handpiece to create easy access for caries excavation. During the removal of unsupported enamel, care was taken to avoid contact with the dentine caries on the internal walls and floor of the cavity. Soft loose debris present over the cavity was then removed. The identification of infected dentine was performed based on colour and consistency [18]. Excavation was performed with sterile sharp spoon excavators (Ash, G5-Claudius Ash Ltd, Potters Bar, Herts, UK) until all parts of the lesion that were light brown and of soft consistency (probe penetrating dentin with no resistance when the probe is removed) were removed. The dentine caries was kept moist throughout the excavation process to minimize over-excavation [19]. The excavated infected dentine samples were placed in a sterile 1.5-mL microcentrifuge tube and transported to the laboratory for further analysis.

### 2.3. Strain Identification

Matrix-assisted laser desorption/ionization time-of-flight (MALDI-TOF) mass spectrometry (MS) was used to identify the strain C10B. The sample preparation for MALDI-TOF MS analysis was carried out as reported previously by Mellmann *et al.* [20]. Firstly, fresh culture of C10B on LB agar was smeared on a MSP 96 target polished steel BC plate and overlaid with 1 μL of MALDI matrix [21] and the sample was air-dried before further analysis using the Microflex MALDI-TOF bench-top MS (equipped with UV laser at wavelength of 337 nm). The method for analysis of the sample was conducted as previously described [21] and the identity of the sample was evaluated based on a dedicated scoring system where the spectra information of the sample was compared to the best match in the Bruker database. The scoring value used was according to those described by Robson and colleagues [21] and a dendrogram was constructed from the standard MALDI Biotyper MSP creation method and generated by similarity scoring of a set of mass spectra illustrating the graphical distance values between species constructed from their MALDI-TOF reference spectra.

### 2.4. AHLs Extraction

Strain C10B was cultured in LB broth buffered with 50 mM 3-(*N*-morpholino) propanesulfonic acid (MOPS) (pH 5.5) and grown by shaking in an incubator shaker (200 rpm, 37 °C, 18 h). An organic solvent, namely acidified (0.1%v/v glacial acetic acid) ethyl acetate, was used to extract the culture twice, as described previously [22]. The organic solvent was dried in a fume hood and resuspended using the same solvent and dried again. After that, 200 μL of acetonitrile (HPLC grade) was added and the mixture vortexed to dissolve the dried extracts completely. The mixture was centrifuged at maximum speed for 5 min to remove any insoluble residue and 75 μL of dissolved sample was aliquotted from the upper layer and placed in sample vials for mass spectrometry analysis.

### 2.5. AHLs Profiling by Mass Spectrometry (MS) Analyses

High resolution MS was performed as previously described by Ortori and coworkers [22] using an Agilent 1290 Infinity LC system coupled together with an Agilent ZORBAX Rapid Resolution High Definition SB-C18 column (50 mm × 2.1 mm, 1.8 μm particle size), with its temperature, flow rate and injection volume as described previously [16]. The mobile phases A and B used in this study were water and acetonitrile (both mobile phases added with 0.1%v/v formic acid), respectively and set to a ratio of 80:20. The parameter for high resolution electron spray ionization mass spectrometry (ESI-MS) set for the run was as described previously [21]. The precursor ion scan mode targeting the *m/z* 102 product ion that indicates that [M + H]^+^ ion of the lactone ring. The *m/z* value range to detect the precursor ions was set at 150–400 and the MS data analysis was done using Agilent MassHunter software.

## 3. Results and Discussion

### 3.1. Bacterial AHLs Preliminary Screening

**Figure 1.**
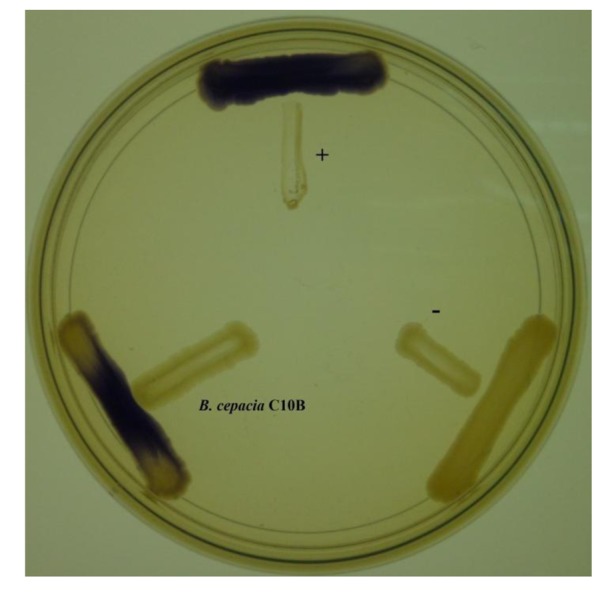
AHL screening of strain C10B with CV026. *E. carotovora* PNP22 (negative control “−”) devoid of QS activity was included and *E. carotovora* GS101 (positive control “+”) that can activate CV026 was included for comparison.

The discovery of a variety of bacterial biosensors have facilitated research on understanding the bacteria cell-to-cell communication system and is able to provide a fast and easy way of detecting bacterial signaling molecules. The luxI/luxR system is commonly employed by proteobacteria where the luxR protein displays specificity towards the cognate AHL molecules and positively regulates a reporter gene transcription cascade [23,24]. The C10B isolate was cross-streaked with *C. violaceum* CV026 to detect the presence of AHL molecules. The strain C10B induced purple violacein pigmentation (Figure 1), indicating that this strain produces short chain AHLs.

### 3.2. Characterization of Strain C10B

**Figure 2.**
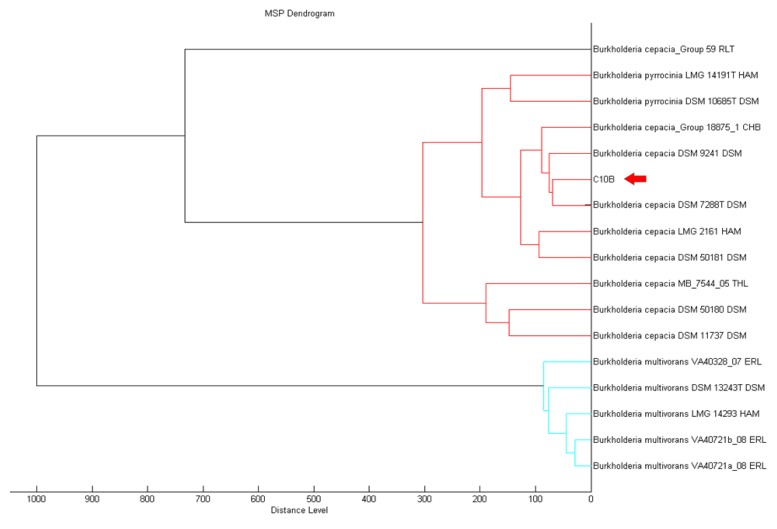
Score-orientated dendrogram that shows the classification of strain C10B. Bacterial strain C10B was clustered hierarchically as *Burkholderia cepacia* (red arrow) based on the protein mass spectra patterns.

MALDI-TOF serves is a reliable method that provides high throughput for the classification and identification of microorganisms in a shorter turnover time [25,26]. MALDI-TOF MS can support rapid and promising means to accurately identify microorganisms [27]. Using MALDI-TOF, strain C10B was best matched as *Burkholderia cepacia* (score value of 2.335) filtered by the MALDI-TOF Biotyper Real Time Classification software. The MALDI Biotyper MSP software further supported the closest match to *B. cepacia* (Figure 2).

### 3.3. AHL Profiling by High Resolution Tandem Liquid Chromatography Mass Spectrometry

*Burkholderia cepacia* is an opportunistic pathogen that primarily infects immunocompromised patients with cystic fibrosis, chronic granulomatous disease, burns or patients with indwelling devices and cancer [27]. This cystic fibrosis-causing agent is believed to possess QS properties as a mechanism to regulate virulence factors in order to express its pathogenicity [27]. Many strategies are being implemented to prevent the outbreak of this disease and the discovery of its QS properties could be one of the strategies to inhibit its virulence.

**Figure 3.**
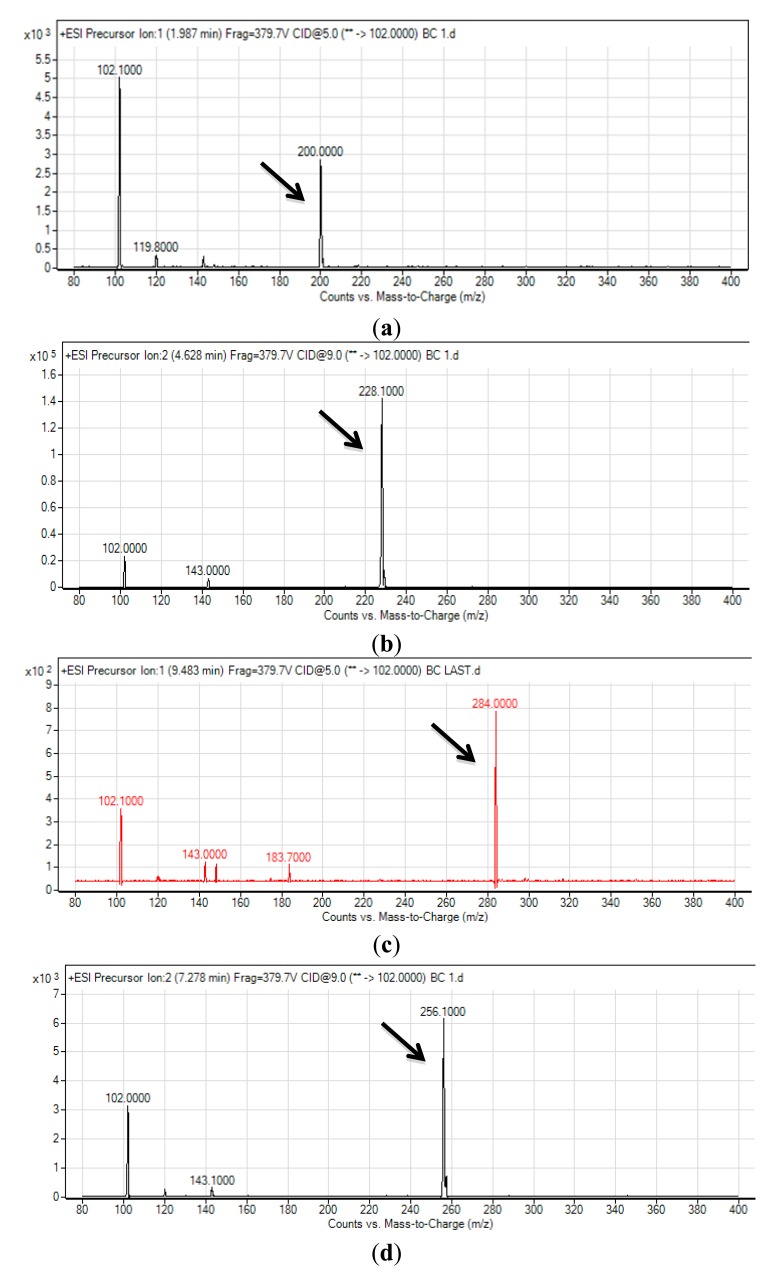
Mass spectrometry analysis of spent supernatant extract of *Burkholderia* sp. strain C10B. All corresponding *m/z* for respective AHLs are marked by arrows; (**a**): mass spectrum of C6-HSL (*m/z* 200.0000), (**b**): mass spectrum of C8-HSL (*m/z* 228.1000), (**c**): mass spectrum of C10-HSL (*m/z* 256.1000), (**d**): mass spectrum of C12-HSL (*m/z* 284.1000).

The spent culture supernatant of C10B strain was analyzed using mass spectrometry (MS) and this confirmed that C10B produced four types of AHL molecules; which are *N*-hexanoyl-L-homoserine lactone (C6-HSL), C8-HSL, *N*-decanoyl-L-homoserine lactone (C10-HSL) and *N*-dodecanoyl-L-homoserine lactone (C12-HSL) (Figure 3). Thus, to our best knowledge, it is believed that the AHL profile shown by strain C10B had been documented for the first time by our group.

Since most *B. cepacia* strains produce two AHLs namely *N*-octanoyl-L-homoserine lactone (C8-HSL) and *N*-hexanoyl-L-homoserine lactone (C6-HSL) [28], we set out to study the AHL profile of our isolate. It has been reported that a soil *Burkholderia* sp. strain GG4 produced multiple AHLs namely 3-oxo-C6-HSL, C8-HSL, 3-hydroxy-C8-HSL and C9-HSL [29], making its AHLs profile very different from that of our isolate. Members of *Burkholderia* are ubiquitous and can be found in many different environmental habitats, ranging from water, soil, water, plants and animals to humans. Phylogenetically, *Burkholderia* can be divided into two major clusters, one that is primarily represented by plant, animal and human pathogenic *Burkholderia* spp. including the opportunistic human ones referred to as *Burkholderia cepacia* complex (BCC) [30]. The second major cluster, that is phylogenetically distant from the BCC, consists mainly of novel environmental, plant-associated, rhizospheric and/or endophytic *Burkholderia* [29,31,32]. Our isolated *Burkholderia* sp. strain C10B showed a very distinctive AHL production profile, and hence opens the scope of QS research on *Burkholderia.*

There are also other bacteria clustered in the *Burkholderia* which are pathogenic against humans and animals. Some *Burkholderia* spp. are reported to use QS to regulate their virulence factors and hence express pathogenicity. One of them is *Burkholderia pseudomallei*, that causes human and animal melioidosis [33]. It has been reported in the work by Ulrich and colleagues that *B. pseudomallei* synthesized multiple AHL molecules, including *N*-octanoyl-homoserine lactone, *N*-decanoylhomoserine lactone, *N*-(3-hydroxyoctanoyl)-L-homoserine lactone, *N*-(3-hydroxydecanoyl)-L-homoserine lactone and *N*-(3-oxotetradecanoyl)-L-homoserine lactone. The parallel between the study done by Ulrich and colleagues with our study is that the pathogenic *Burkholderia* sp. produces multiple AHL molecules that could modulate pathogenicity towards humans and animals. Another member of *Burkholderia*, *Burkholderia mallei*, the causative agent of the equine disease glanders employs QS as a regulatory system to express its pathogenicity [34]. Majerczyk and colleagues reported that the pathogenic *B. mallei* strain produced two types of AHL molecules which are *N*-octanoyl-homoserine lactone and *N*-decanoyl-homoserine lactone. These findings demonstrate that members of *Burkholderia* carry multiple *luxIR* homologs that either directly or indirectly regulate the biosynthesis of essential virulence factors that contribute to the *in vivo* pathogenicity of *Burkholderia* [35]. It appears that *Burkholderia* members seem to show varying AHL production profiles but no clear pattern is observable.

QS regulates a battery of physiological activities in bacteria such as motility and biofilm formation. This work illustrated the significance of research on AHL-producing human pathogens present in the host environment. This finding also aids in providing an alternative solution in preventing disease outbreaks, as well as to understand the pathogen in more depth.

In addition, reporting from this study provides an insight that dentine caries having access to the oral cavity could be a potential reservoir for QS pathogens and hence intense research should be applied to further study the presence of *Burkholderia* sp. in the human body.

## 4. Conclusions

We have reported the unusual multiple production of AHLs by *Burkholderia* sp. strain C10B isolated from dentine caries and to the best of our knowledge, this is the first example where *Burkholderia* sp. produced four types of AHL (C6-HSL, C8-HSL, C10-HSL and C12-HSL).
